# Multiple air-bubble enhanced oil rupture on nanostructured cellulose fabric for easy-oil cleaning fouled in a dry state

**DOI:** 10.1038/s41598-019-51216-7

**Published:** 2019-10-10

**Authors:** Min-Sung Kim, Tae-Jun Ko, Seong Jin Kim, Young-A. Lee, Kyu Hwan Oh, Myoung-Woon Moon

**Affiliations:** 1 Department of Materials Science and Engineering, Seoul National University, Seoul, 08826 Republic of Korea; 2 Materials and Life Science Research Division, Korea Institute of Science and Technology, Seoul, 02792 Republic of Korea; 3 NanoScience Technology Center, University of Central Florida, Orlando, Florida 32826 USA

**Keywords:** Structural properties, Environmental, health and safety issues

## Abstract

Nanostructured cellulose fabric with an air-bubble-enhanced anti-oil fouling property is introduced for quick oil-cleaning by water even with the surface fouled by oil before water contact under a dry state. It is very challenging to recover the super-hydrophilicity because once the surface is oil-fouled, it is hard to be re-wetted by water. Anti-oil-fouling under a dry state was realized through two main features of the nanostructured, porous fabric: a low solid fraction with high-aspect-ratio nanostructures significantly increasing the retracting forces, and trapped multiscale air bubbles increasing the buoyancy and backpressure for an oil-layer rupture. The nanostructures were formed on cellulose-based rayon microfibers through selective etching with oxygen plasma, forming a nanoscale open-pore structure. Viscous crude oil fouled on a fabric under a dry state was cleaned by immersion into water owing to a higher water affinity of the rayon material and low solid fraction of the high-aspect-ratio nanostructures. Air bubbles trapped in dry porous fibers and nanostructures promote oil detachment from the fouled sites. The macroscale bubbles add buoyancy on top of the oil droplets, enhancing the oil receding at the oil-water-solid interface, whereas the relatively smaller microscale bubbles induce a backpressure underneath the oil droplets. The oil-proofing fabric was used for protecting underwater conductive sensors, allowing a robot fish to swim freely in oily water.

## Introduction

Oil is expected to remain an important source of energy as oil-related industries such as mining, transportation, and refining continue to grow despite the emergence of several alternative energy sources over the recent decades^1^. A greater demand for oil-spill management and efficient oil–water separation technologies is also expected because oil spill accidents occur in large sizes and in a wide range of locations. However, technologies dealing with oil spill responses still depend on conventional methods that have yet to overcome the limits of low-efficiency, high cost, and human-dependent control activities^2–6^.

To separate or remove spilled oil in water, sorbents, filters, or membranes having either hydrophobic/oleophilic or hydrophilic/underwater oleophobic surfaces have been intensively studied^7^. Among them, hydrophilic and underwater oleophobic materials have been heavily used recently because a hydrophilic surface is easily water-wetted, upon which the oil is repelled by water. Such materials have several advantages in various applications such as self-cleaning, anti-fogging, biocompatible materials, oil transportation, or oil-water separation^8–11^. Additionally, oleophobicity and self-cleaning behavior can also be provided by superhydrophilic surfaces in the wetted state by water^11,12^. The significant disadvantage of existing superhydrophilic surfaces, however, is that they could be easily fouled by oil because of their high surface energy, thus losing their super-wetting characteristic^13–16^. It is also hard to re-wet by water and lose underwater oleophobicity due to the pre-absorbed oil that is hardly removed by water. Additionally, it was reported that once the textured surface is wetted by oil, it is very challenging to recover its super-hydrophilicity due to the strong capillary interactions between oil and the textured surfaces^13,16^. Once fouled, especially on a structured surface with nanopores, the wetting transition from the Cassie-Baxter state to the Wenzel state is typically irreversible^13^, thus limiting their use in practical applications. Significant energy or work is necessary to overcome capillarity to push the infiltrated liquid out of the nanopores^13,17^. An unstable surface will decrease in terms of work efficiency during the oil–water separation process, and increase in cost, which requires a certain level of protection from oil contamination during use. Recent studies have presented anti-oil fouling hydrophilic surfaces under a dry state using a coating of zwitterionic polyelectrolyte^16,18–21^. Surface coating using a polycrystalline TiO_2_ thin film with ultra-violet irradiation or SiO_2_-nano-particle added low-surface-energy polymers have also been reported to show an oleophobic nature in both air and underwater^22,23^. However, to use coating-based materials, such attempts require mechanical or chemical durability for many cycles during long-term use^9^.

**Figure 1:**
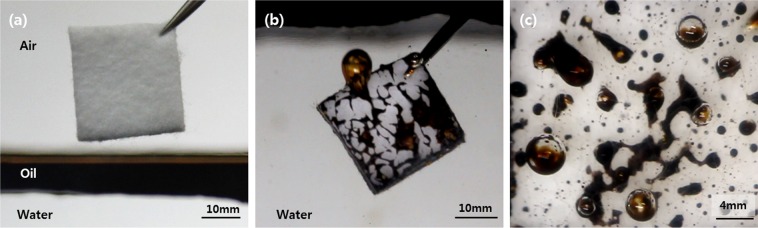
(**a**) Dry rayon immersed in oil-covered water, (**b**,**c**) air bubbles retained in fabric pulling oil residue in water.

Herein, we present an easy-oil cleaning porous fabric made of hygroscopic, hydrophilic cellulose with nanostructures, which remains so even if oil-fouled before contact with water without any functional coating. Easy-oil cleaning by water was conducted by significantly increasing the retracting forces using nanostructures with a high aspect ratio (AR), the ratio of height over the width of the nanostructures, as well as through assistance from the air bubble, inducing oil mobility on the fibers. The buoyancy and backpressure induced in water recovered the wetting transition from the Wenzel (oil-fouled) state to the Cassie-Baxter (water cleaned) state reversibly. Selective oxygen plasma etching was used to provide a high AR for nanostructures and chemically hydrophilic hydroxyl groups, which strongly renders the hydrophilic fabric surfaces superhydrophilic, having the water contact angle near zero degrees in air. It was found that treated, oil-fouled fabric in a dry state is easily cleaned through immersion into water, as shown in Fig. 1 (also see Movie S1). When a dried fabric is immersed into oil-covered water (Fig. 1a), the submerged fabric is initially fouled by oil at the oil–water interface, and the oil starts to recede on the fabric surface. During the oil receding process, air bubbles are observed at multiple sites of the fabric, lifting the oil residue (Fig. 1b). The air retained inside the porous fabric in a dry state are trapped by the oil covering during immersion into oily water, and as the covered oil is receded, air bubbles will also escape the porous fabric by pulling the oil droplets (Fig. 1c). The non-woven fabric has random roughness surface which hardly maintain the exact equal experiment environment. To obtain the reliable data, we have conducted experiments more than three times. To investigate the anti-oil fouling characteristic even with oil fouling before water contact, we have conducted experiments for surface roughness enhancement, the air bubble effect, backpressure effect, cyclic test for durability, and practical application. The wetting property was controlled by two factors, chemical composition and roughness of the surface^24^. Oxygen plasma treatment enhanced the two factors simultaneously for hydrophilicity and underwater oleophobicity. The effect of oxygen plasma treatment was investigated in surfaces treated for up to 60 min and compared with the pristine (not treated) surface. The surface roughness was investigated by scanning electron microscopy (SEM) and Image J software. The oxygen plasma-treated surfaces were fouled by crude oil under the dry state and the oil receding behaviors were observed in the underwater state. The air bubbles induced by the oil-fouled fabric were observed and their effect on the oil receding behavior at underwater environment was evaluated. To evaluate the air bubble effect, we prepared pristine fabric and a 10 min oxygen plasma-treated surface that were also fouled by crude oil and immersed in water. Then, for each case, the induced air bubbles were removed using an oil absorbent. After the air bubble effect experiment, we also investigated the backpressure effect that was controlled by the thickness of the fabric. The prepared fabrics immersed in oil were covered by water. The fouled oil was observed to rupture and recede on the surface of the fabric assisted by air bubbles. We also conducted a cyclic immersion test for the duration of the underwater oleophobicity. Finally, we demonstrated a practical application, namely, an oil-proofing sensor cover for underwater uncrewed vehicles.

We explored the effects of nanostructures and air bubbles on the oil cleaning of a cellulose fabric under a dry state. Furthermore, we conducted a cyclic immersion test, showing that the nanostructured cellulose surface, oil-fouled under a dry state, is continuously cleaned during cyclic immersion, whereas a pristine fabric without a nanostructure remains fouled on most of its surface. Finally, we applied an anti-oil fouling fabric as an underwater-sensor protective fabric to an oil-fouling operation in an oil-spilled environment.

## Results and Discussion

### Oil cleaning on nanostructured hydrophilic fabric

**Figure 2:**
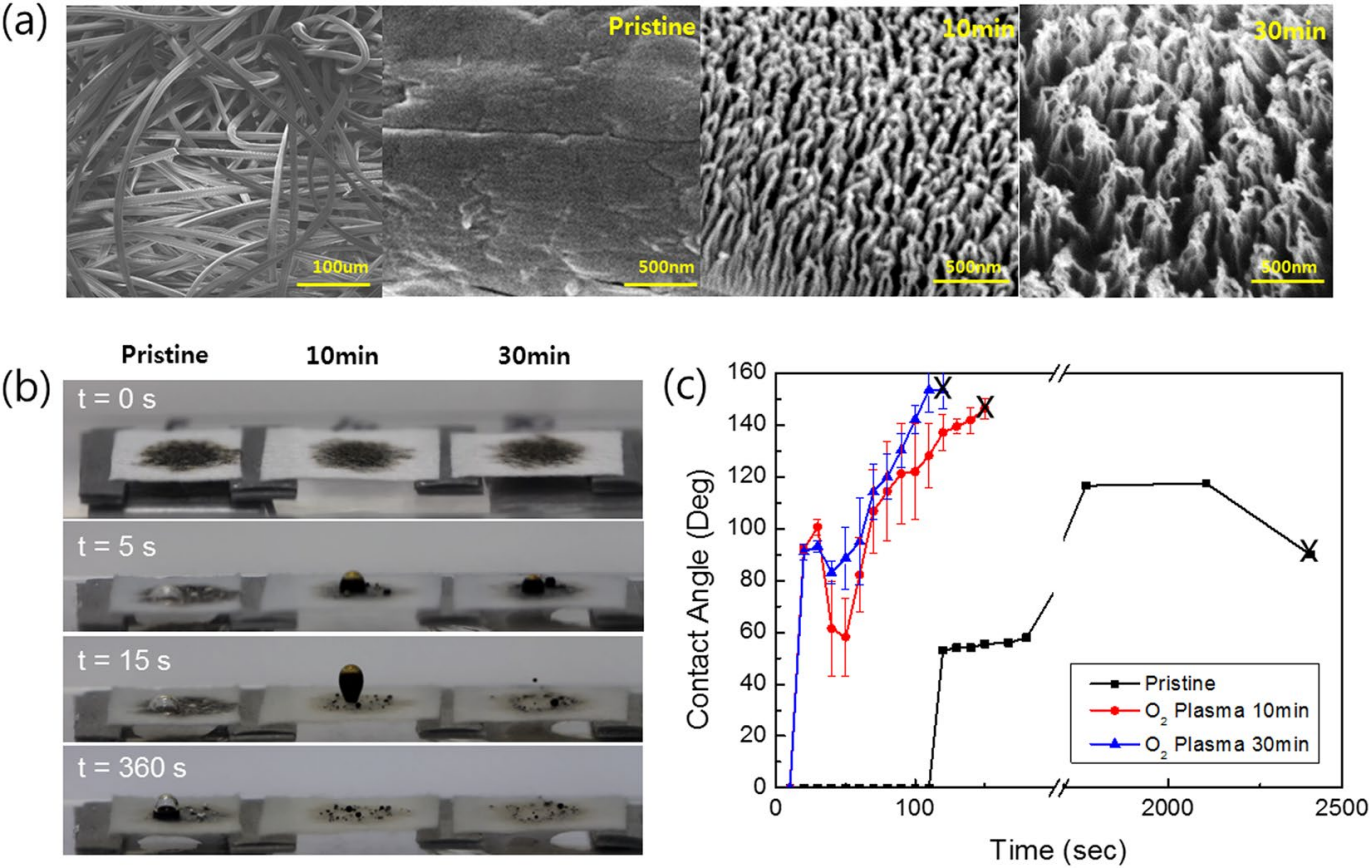
(**a**) SEM images of the surface morphology for surfaces of pristine non-woven rayon fabric and nanostructured surface with O_2_ plasma treatment of 10 and 30 min. (**b**) Oil releasing behavior on the pristine and nanostructured surface in water and (**c**) oil contacting angle traced in water over time.

Rayon fabrics, made from regenerated cellulose, were prepared under six different treatment conditions by choosing the oxygen plasma treatment duration, namely, no treatment (pristine), 1 min, 5 min, 10 min, 30 min and 60 min treatments, the results of which showed that the measured ARs of the nanostructures were 1.3, 8.0, 11.3, 37.2, and 55.9, respectively. A reduction in oil detachments was observed at 514, 311, 153, 124, to 135 s, respectively. Even though oil detachment was also observed on the surfaces treated by oxygen plasma for 1 (AR 1.3) and 5 min (AR 8.0), it was relatively slower than that in the other cases, where it occurred between 10 and 60 min. Therefore, the oil-repelling ability or speed was enhanced sufficiently after 10 min plasma treatment or an AR of 11. as shown in Fig. 2a. It was reported that the nanostructures were fabricated through the metal-incorporated dry ion etching process used in plasma treatment^25^. During the plasma treatment, metal particles are sputtered from the cathode plate and then co-deposited onto the rayon surface. These metal particles act as an etching mask upon which the polymer surface shows a low etching rate for oxygen plasma. However, the regions where these metal particles are not deposited have a high etching rate depending on the plasma treatment duration, and thus are easily etched, eventually forming a high AR nanostructure^25^. The oil contact angle and detachment time were measured in water (Fig. 2b,c) on the three different fabric surfaces. The pristine rayon fabric fouled by oil droplets in a dry state was immersed in water, and the oil residue started to recede and increased the oil contact angle after 110 s, fully detaching with an oil contact angle of approximately 110° after 40 mins. It was observed that the pre-wet rayon fabric has a strong oil repellent nature in comparison to the dry fabric, as shown in Fig. S1, owing to the presence of a water film between the oil and rayon. In contrast, a nanostructured rayon fabric fouled by oil droplets in a dry state (30 min duration, AR of ~37) showed a relatively faster oil detachment by increasing the oil contact angle up to 157° after 100 s, reaching full detachment at 2 min (see Fig. 2c). It was found that in all cases of pristine and nanostructured surfaces, air bubbles that had formed on top of the oil droplets were observed to pull the oil residue off of the fabric. It can be presumed that the air bubbles contributed to the oil receding and detachment by adding additional upward forces to the oil drops against the interface adhesion strength between the oil and fabric surface.

### Nanostructure-enhanced oil detachment with air bubbles and backpressure

**Figure 3:**
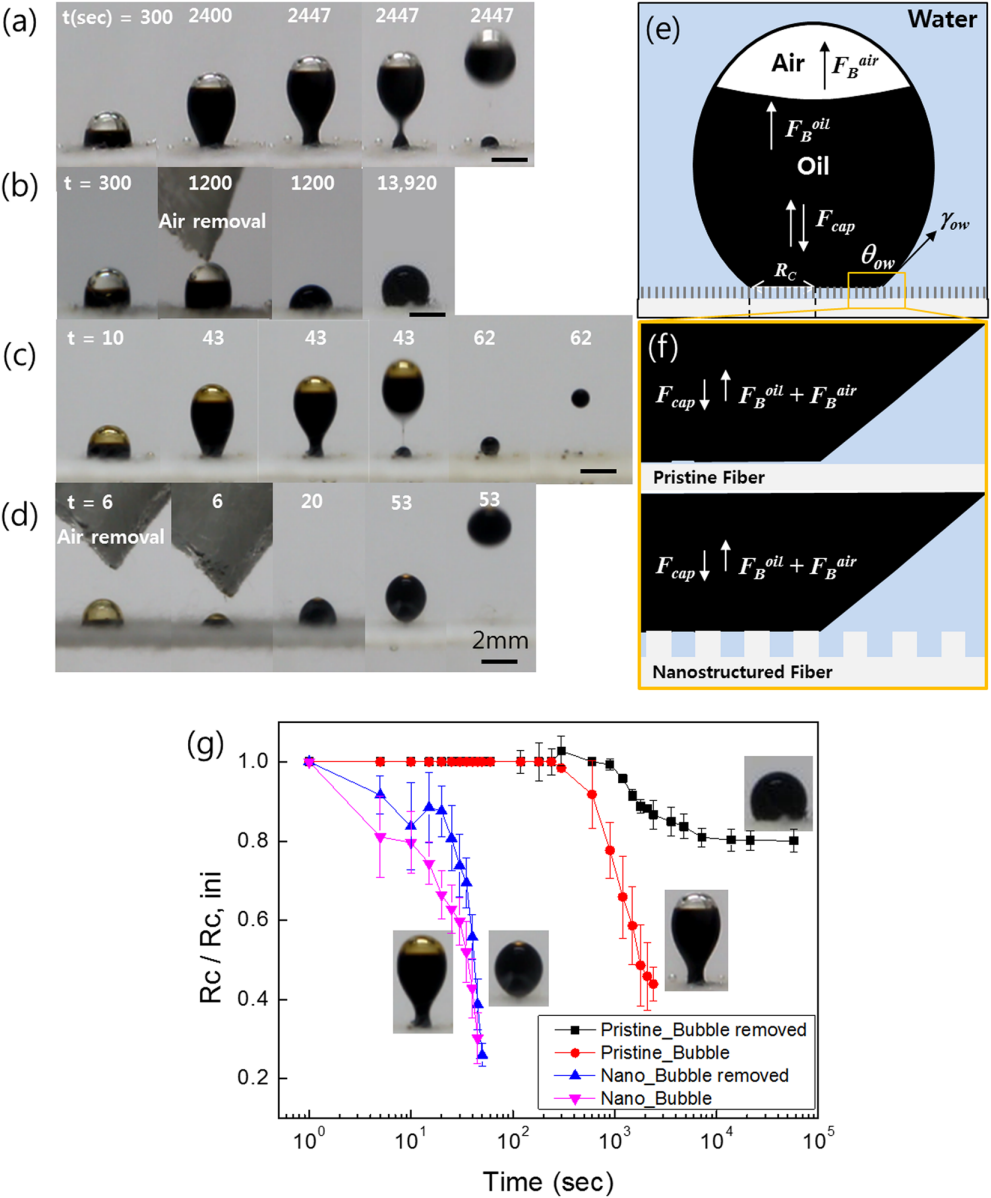
Sequential images of oil receding on rayon surface with/without bubbles and nanostructure in water over time. Oil droplet on the pristine rayon surface (**a**) with and (**b**) without air bubbles, and a nanostructured surface (**c**) with and (**d**) without air bubbles. Oil receding model with forces acting near oil droplet on nanostructured surface in water. (**e**,**f**) Schematic of force balance of oil and air bubbles. (**g**) Ratio of oil contact radius (*R*_*c*_) to the original oil contact radius (*R*_*c*−*ini*_) with/without air bubbles and nanostructure fabric.

**Figure 4:**
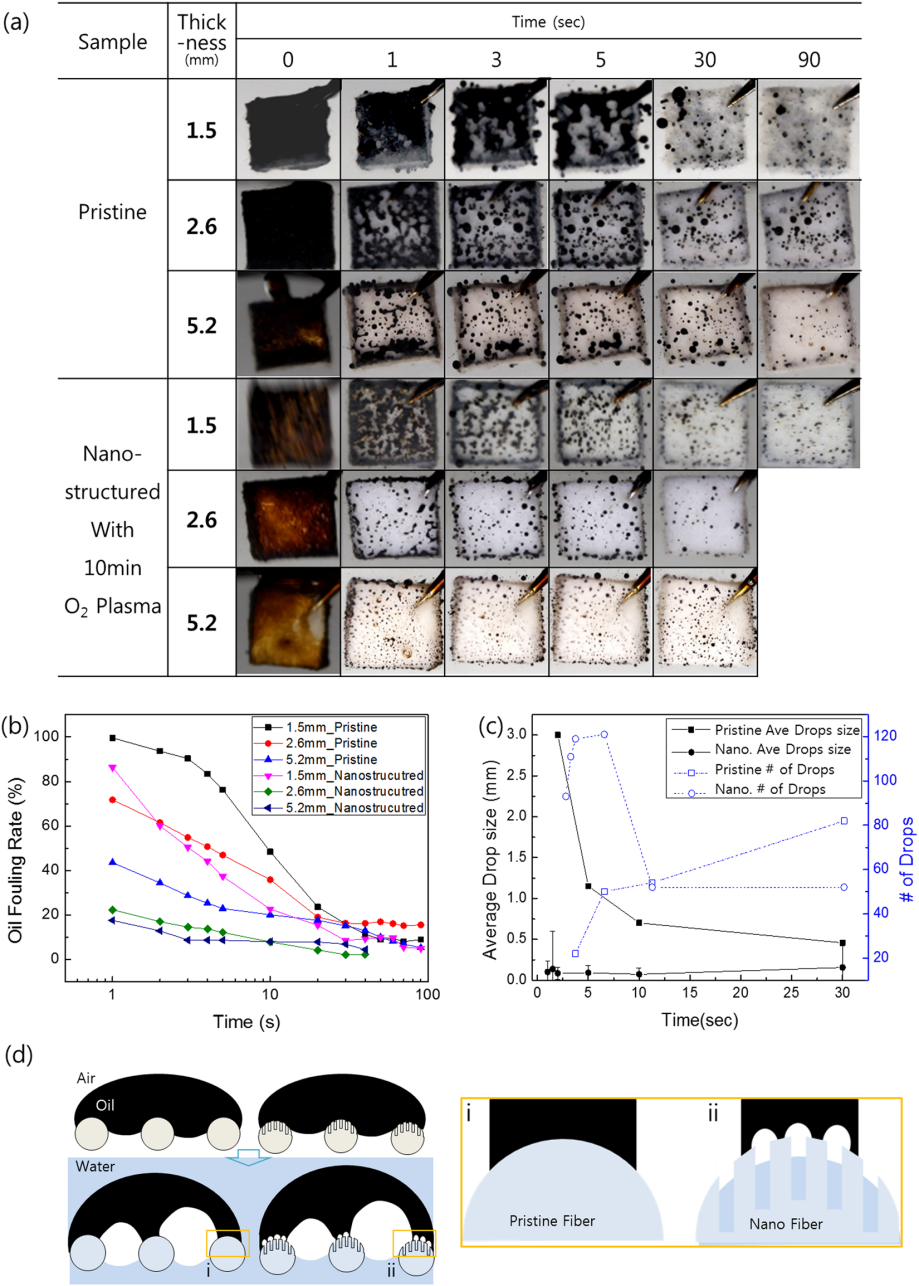
(**a**) Optical sequential images for oil cleaning on fabrics with and without nanostructures for three different thicknesses. (**b**) Oil fouling ratio for thicker sample having a steeper slope than that for the thin sample. Furthermore, the nanostructured samples are further enhanced in terms of the oil fouling rate for both thicknesses. (**c**) Pristine surface without nanostructures and relatively larger oil residues. In contrast, in the case of the nanostructured fabric, smaller oil residues were observed. (**d**) Schematic of air-bubble induced process on pristine and nanostructured fabrics.

To explore the role of air bubbles on the oil detachment, experiments with and without air bubble on top of the oil drop was conducted, as shown in Fig. 3. First, the dry rayon fabric was fouled by 10 μl of crude oil in an air environment, and placed into water to compare the underwater receding behavior of the triple contact line of oil/water/solid for the different surface conditions. The oil detachment force can be described by the following equation as schematically shown in Fig. 3e:

1 $${F}_{detach}={F}_{B}^{oil}+{F}_{B}^{air}+{F}_{cap},$$

where *F*_*B*_^*oil*^ is the oil buoyancy (=(*ρ*_*water*_ − *ρ*_*oil*_)*gV*_*oil*_), *F*_*B*_^*air*^ is the air buoyancy (=(*ρ*_*water*_ − *ρ*_*air*_)*gV*_*air*_), *ρ* is the fluid density, *g* is the gravitational acceleration, and *V* is the fluid volume. *F*_*cap*_ is the capillary force acting at the contact line and is estimated as

2 $${F}_{cap}=2\pi {R}_{c}{\gamma }_{ow}(\cos \,\theta -\,\cos \,{\theta }_{s}),$$

where *γ*_*ow*_ is the interfacial tension between oil and water, and *θ* and *θ*_s_ are dynamic and static contact angles of an underwater oil droplet on the fabric, respectively. Note that *F*_*cap*_ is positive when *θ* < *θ*_s_, which act_s_ to detach the oil, while it is negative when *θ* > *θ*_s_, which plays the role of capillary adhesion that resists oil detachment. For the pristine fabric, the air bubbles were observed to have a significant effect, especially in a later stage of the oil detachment, by pulling the oil up through the air buoyancy of *F*_*B*_^*air*^ ≈ 36 μN along with the receding capillary force. e.g. *F*_*cap*_ ≈ 15 μN at 2400 s, where a *θ*_*s*_ of 129° is used on the pristine fabric (Fig. 3a). This is contrary to the case without air bubbles in which *F*_*B*_^*air*^ is nearly zero because the air bubble is removed when it is pricked with a hydrophobic absorbent (Fig. 3b)^26^. These air bubbles arise from the micro-pores of the fabric owing to buoyancy after the oil had covered these pores. Subsequently, as shown in Fig. 3a, the oil droplets are elongated vertically, and meanwhile, the contact line recedes at a low speed. This temporal behavior is plotted in Fig. 3g using the ratio of the oil contact radius (*R*_*c*_) to the initial conta_*c*_t radius (*R*_*c-ini*_). When the contact radius decreases to a specific value (*R*_*c*_/*R*_*c-ini*_ ≈ 0.4), the oil column near the solid surface is rapidly pinched off after 2,447 s owing to vigorous capillary pumping out of the necked location^27,28^. The oil remaining below the pinch-off location does not recede further because it lacks air bubbles to be drive the receding oil. In other words, *F*_*B*_^*oil*^ (≈0.4 μN) and *F*_*cap*_ (≈35 μN) of the remaining oil are not enough to further cause oil detachment by overcoming the viscous adhesion (*F*_*vis*_) of the oil fouling between the fibers. In contrast, an oil droplet with no air bubble (Fig. 3b) shows extremely slow retraction of the contact line on pristine rayon in water, which indicates the effect of air buoyancy on the receding contact line. The oil then recedes at a low speed for nearly 4 h (≈13,920 s) and stops, further receding when it reaches the static contact angle because the oil buoyancy (≈8 μN) is insufficient to overcome the capillary adhesion as well as the viscous adhesion,

Nanostructured cellulose fabric (Fig. 3c,d) presents an essential difference in the oil receding performance in comparison to pristine fabric. Regardless of whether air bubbles are present, the oil droplets were removed from the solid surface within ≈1 min. The static contact angle does not remain stable. Instead, it diverges as *θ* → 180° on the nanostructured cellulose fabric. Thus, *F*_*cap*_ is always positive on the nanostructured fabric and is supposed to detach the oil with a stronger force than that acting on the pristine fabric. Additionally, the relatively minor difference caused by the air bubbles is that the oil droplets with air bubbles initially recede faster than those without air bubbles owing to additional air buoyancy, and then pinch off quite quickly at 43 s, with a residual amount left on the solid surface (Fig. 3c). However, unlike the pristine case, the residual oil further recedes and detaches from the solid surface for the nanostructured fabric. The remarkable performance of the nanostructure over the air buoyancy originates from the partial contact of oil with a hydrophilic nanostructure, which is in contrast with the full contact on the pristine fiber, as schematically compared in Fig. 3f. This partial contact contributes to boosting the receding contact line by enhancing *F*_*cap*_ from diverging *θ*_*s*_ as well as alleviating the viscous adhesion (*F*_*vis*_) of oil fouling between the fibers, which can be quantified by measuring the contact angle hysteresis (CAH)^29–33^ The extreme difference in CAH between the pristine and nanostructured surfaces is demonstrated in Supplementary Movie 2, which shows that an oil droplet is firmly stuck to pristine rayon even up to a sliding angle of ≈90° in an underwater environment. In contrast, the oil on nanostructured rayon easily rolls off at a sliding angle of ≈20°, which contrasts the superior property of the low CAH of nanostructured rayon. This low CAH is caused by partial contact between oil and a solid fiber with a nanostructure, which is referred to as the Cassie–Baxter state^30^. The Cassie–Baxter state on the nanostructure also implies that the air-pocket is capable of sticking between the nano-pillars of the rayon even when fouled by oil in a dry environment. Later, when put into water, this trapped air further pushes off the oil drop on top of the nanostructure as the water wicks through the fabric with the pushing air. This air beneath the oil droplet is known to act as a backpressure with improved liquid mobility by inducing a de-pinning of the oil^34–36^, as schematically shown in Fig. 4d. For the nanostructured rayon fiber to have a high AR, the air backpressure acts at numerous locations even on a single fiber, which is thought to essentially boost the de-pinning by significantly reducing the de-pinning distance down to the nano-scale pitch between adjacent nano-pillars.

### Air-bubble induced oil cleaning

Here, to further explore the air-bubble effect on the oil detachment, we varied the thickness of the fabric, which equivalently changed the total amount of air trapped in the microporous fiber network. The thicknesses of the prepared fabrics were 1.5, 2.6, and 5.2 mm, as shown in Fig. 4a. It can be seen that the oil immediately fouled all surfaces in air, and then receded with a different detachment behavior over time underwater. The oil receding time is shorter for the thicker sample (5.2 mm) in comparison with the thinner sample (1.5 mm). The thickness effect is more dramatic in comparison with the samples after 10 min of O_2_ plasma treatment owing to the presence of the nanostructure. Furthermore, the effects of the bubbles can be clearly identified during the early stage. After 1 s of oily water immersion, most of the area for the thin fabric without a nanostructure is fouled by oil, and a larger area was cleaned by bubbles until 90 s. With an increased thickness of the samples, the fouling rate of oil residue shortens and is significantly enhanced with the nanostructures. The oil fouling rate, which is defined as the oil covered area over the total surface area of each sample over time, was traced in water. As indicated in Fig. 4b, the oil fouling ratio measured for a thicker sample has a steeper slope than that for a thin sample. Furthermore, the nanostructured samples are further enhanced in terms of the oil fouling rate for both thicknesses. However, non-woven structures of the fabric trapped some oil droplets, despite the oil receding completely and detaching from the single nanostructured fibers. Fig. S2 shows the oil trapped by adjacent fibers. It has completely receded with an almost spherical shape. In the case of a nanostructured fabric, it was observed that the size of the oil residue is smaller than that of the pristine fabric shown in Fig. 4c. In the case of the pristine surface without a nanostructure, the relatively larger oil residue of approximately 2–3 mm in radius was mainly formed at the beginning of the water immersion test, and the small sized residues increased over time. In contrast, in the case of the nanostructured fabric, a smaller oil residue of approximately 0.1–0.2 mm was observed over multiple sites. It was reported that the oil droplets are small on the nanostructure surface because the energy barrier for rupturing is low owing to the viscous adhesion force, F_vis_, between the oil and solid, which is significantly low from the lower solid fraction^37–39^.

### Cyclic test for self-oil-cleaning

**Figure 5:**
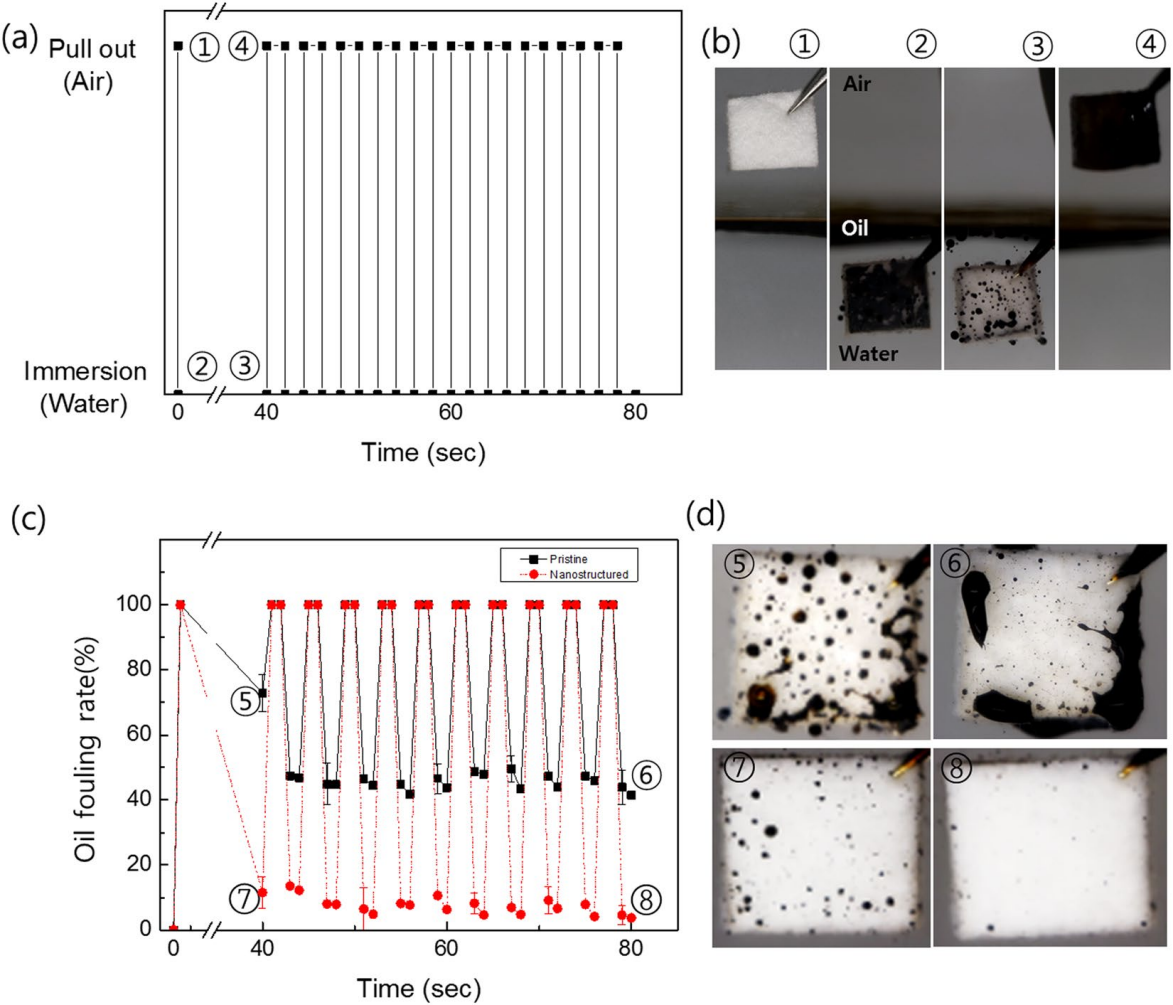
(**a**) Cyclic immersion test condition in oily water for ten cycles and (**b**) representative images of rayon fabric at each position in (**a**) air and water. (**c**) Oil fouling rate measured on the pristine (black squire) and nanostructured (red circle) fabric surfaces and (**d**) its representative images. All samples in (**b**,**d**) are 10 mm × 10 mm in size with a thickness of 2.6 mm.

We further explored the oil detachment behavior on the samples by applying a cyclic immersion test into oily water. A dry rayon sample of 2.6 mm in thickness was immersed in oil covered water, and ten cycles of a pull out/immersion experiment were applied, as shown in Fig. 5a. First, for immersion into oily water (see the representative image ① in Fig. 5b), the dried rayon was immediately fouled (②), and remained for 40 s underwater reaching a steady state to allow the oil to recede (③) from the nanostructured or pristine fabric. The sample was taken out of the water tank and immersed into water again (④). Continuing with this cycle, the oil fouling ratio of the pristine fabric decreased from 73% to 47% (⑤ in Fig. 5c), and then remained at approximately 40% at the tenth cycle (⑥). However, the oil fouling ratio of the nanostructured fabric was measured as 6.5% for the first cycle (⑦), and then maintained an oil residue of around 5% at the tenth cycle (⑧). Representative images of the oil fouling fabric surface in each step (Fig. 5c) were taken, and are shown in Fig. 5d.

### Oil-proofing sensor cover for robot fish

**Figure 6:**
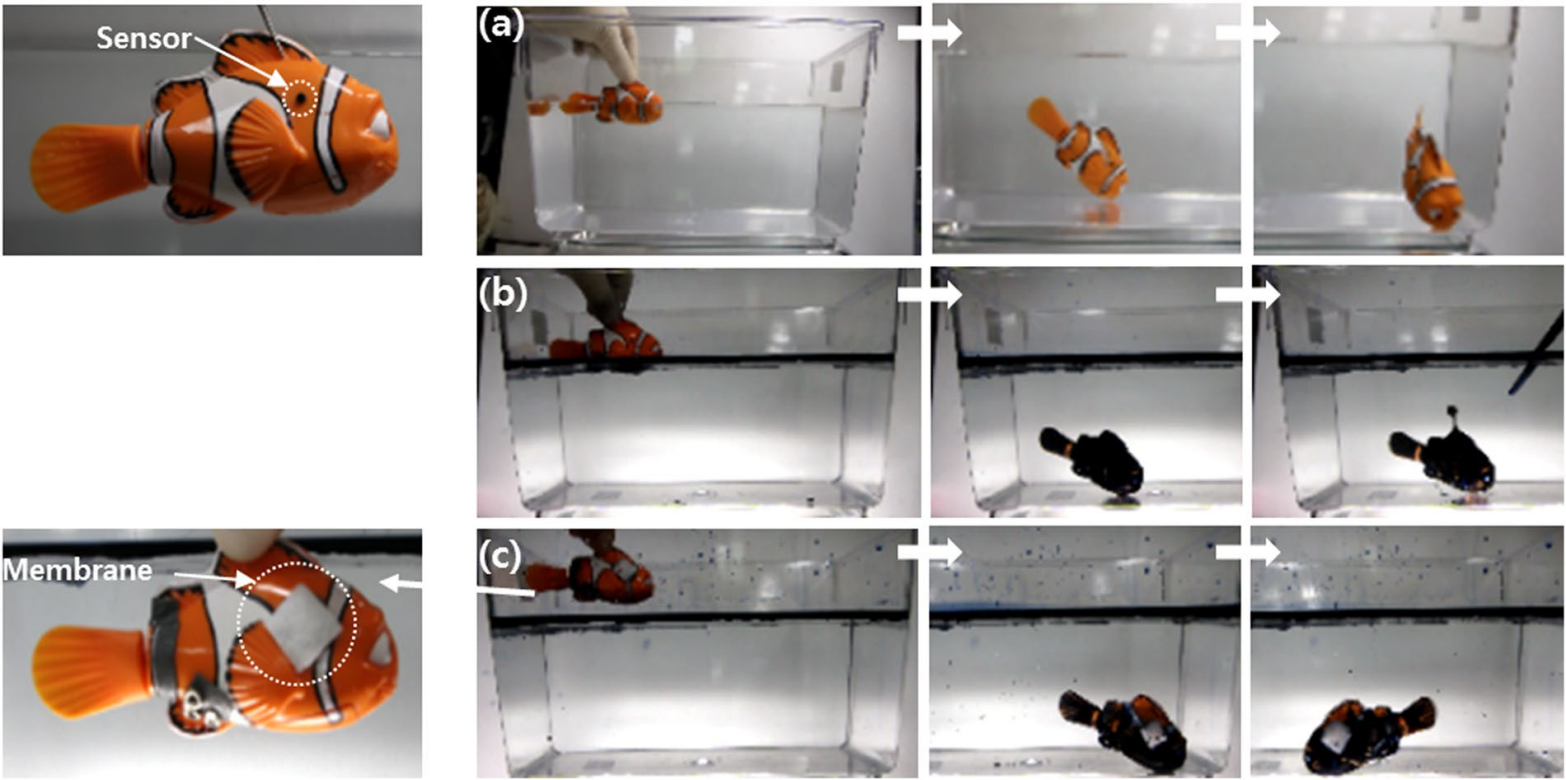
A demonstration of oil-proof or oil-cleaning fabric used as a protective cover on conductive sensors on a robot toy swimming in water. A robot swimming (**a**) in fresh water without fabric, (**b**) in oily water without fabric, and (**c**) in oily water with fabric. The length of the robot toy is approximately 70 mm.

Finally, we applied the fabric to a protective material for the sensing system of a robot fish swimming in oily water. Underwater sensors are used for many different applications, which require several special functions such as anti-bio or mineral fouling. In addition, the sensors working under an oily environment should be equipped with an anti-oil fouling or oil-proofing surface. We covered the eyes of the robot fish with a nanostructured fabric. The robot fish was designed to swim by moving its tail fin when immersed in water. The motor for the moving tail fin is operated by detecting the electronic conductivity through the two eyes in a conductive water environment. If one or both eyes are blocked by a non-conductive substance such as oil, the electronic signal is cut off and the robot fish can no longer move. When immersed in fresh water, the robot fish swims freely, as shown in Fig. 6a (also see Movie S3a). When the robot fish was placed in oily water, it did not move at all owing to the non-conductive oil completely covering both of the sensors, as shown in Fig. 6b (see Movie S3b). However, when the robot fish with the nanostructured fabric was placed into oil-covered water, the eyes were oil-proof but water was allowed to flow through the membrane, allowing the fish to move freely underneath the oily water (Fig. 6c., also see Smovies 3c and 3d).

In conclusion, a quick oil-cleaning surface even under a dry state of oil fouling was suggested for use in cellulose fabric with a high AR nanostructure. The anti-oil fouling under a dry state was realized through two main features: a low solid fraction with high AR nanostructures significantly increasing the retracting forces and number of air bubbles trapped between the fibers and nanostructure under a dry state, thereby increasing buoyancy, and multiple backpressures enhancing the oil layer rupture. The nanostructured fabric was continuously cleaned during cyclic immersion into oily water, whereas the pristine fabric remained fouled owing to the slow oil cleaning of the initial oil fouling. It can be understood that anti-fouling under a dry state can be induced by the air bubbles formed in multiple locations, whereas the anti-fouling under a wet state would be induced by the water coating on the nanostructured surface. Further, we demonstrated the application of oil-proofing fabric for protecting the underwater conductive sensors on a robot fish from oil fouling, while allowing the conductive water to penetrate the sensors. Along with various applications for oil–water separation membranes or cleaning devices, such an oil-cleaning fabric with a nanostructure can be applied to the oil-proof surfaces for several oil-field devices or ships exploring or traveling in oil-contaminated areas.

## Methods

Non-woven rayon fabric, which was hydrophilic and with underwater oleophobicity, were purchased from a commercial company (catalog # SPL80(FC)V, Nam Yang Non-Woven Fabric Co., South Korea). The non-woven type fabric was prepared at a size of 2.5 cm × 2.5 cm and with a density of 158.5 kg/m^3^. Oxygen plasma etching was utilized to form the nanostructures on the rayon fibers using a custom-built plasma etching device having the plasma power and substrate bias voltage set at 230 W and 400 V, respectively^25^. With an oxygen gas flow rate of 100 sccm, the plasma treatment duration applied was 10 and 30 min, resulting in various ARs, namely, 11.4, and 37.4, respectively.

The surface morphology was explored using a SEM (Inspect-F, FEI, USA). A 6-nm thick Pt film was covered onto the fabric to provide better conductivity and prevent the generation of an electron charge on the polymeric fabric. The SEM electron accelerating voltage was 7 kV. The contact angle (CA) of oil was measured using underwater conditions for a light crude oil of 0.87 g/ml in density (Hyundai Oil Bank Co., South Korea) and droplets with a volume of 10.0 ± 0.5 μl. A water immersion test was conducted on various fabrics and recorded with a digital camera with a resolution of 15 megapixels (Canon EOS 60D).

## Supplementary information

Supplementary Information

Supplementary movie 1

Supplementary movie 2

Supplementary movie 3(a)

Supplementary movie 3(b)

Supplementary movie 3(c)

Supplementary movie 3(d)
