# Supplementary material for: Multiple air-bubble enhanced oil rupture on nanostructured cellulose fabric for easy-oil cleaning fouled in a dry state
Source: Sci Rep. 2019 Oct 10;9:14538. doi: 10.1038/s41598-019-51216-7 (PMC6787182; doi:10.1038/s41598-019-51216-7)
Supplement: Supplementary file 1 — Supplementary Information [file 41598_2019_51216_MOESM1_ESM.docx]

**Supplementary Information:**

**Multiple air-bubble enhanced oil rupture on nanostructured cellulose fabric for easy-oil cleaning fouled in a dry state**

Min-Sung Kim^1,2^, Tae-Jun Ko^3^, Seong Jin Kim^2^, Young-A Lee^2^, Kyu Hwan Oh^1^, and Myoung-Woon Moon^2,*^

^1^Department of Materials Science and Engineering, Seoul National University, Seoul 08826, Republic of Korea

^2^Materials and Life Science Research Division, Korea Institute of Science and Technology, Seoul 02792, Republic of Korea

^3^NanoScience Technology Center, University of Central Florida, Orlando, Florida, 32826, USA

*Corresponding Author: [mwmoon@kist.re.kr](mailto:mwmoon@kist.re.kr)


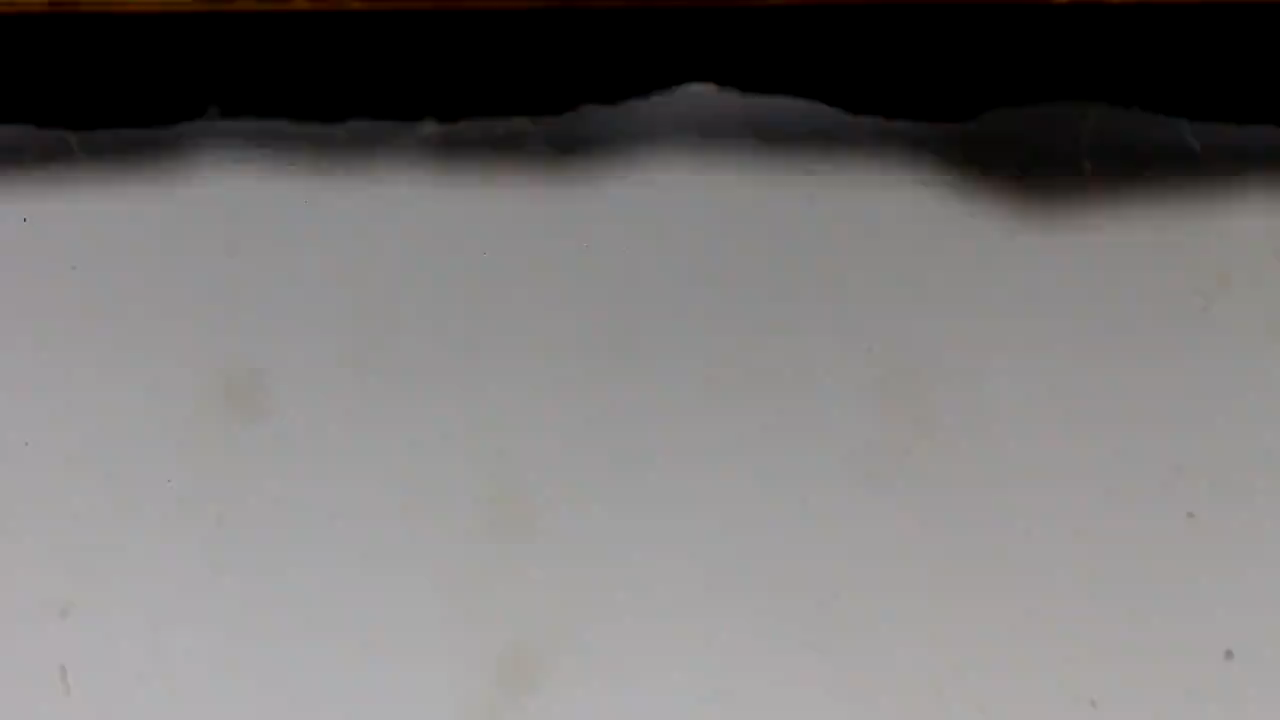


Movie S1. Movie showing oil fouling and oil-cleaning process on dry fabric immersed into oily water.


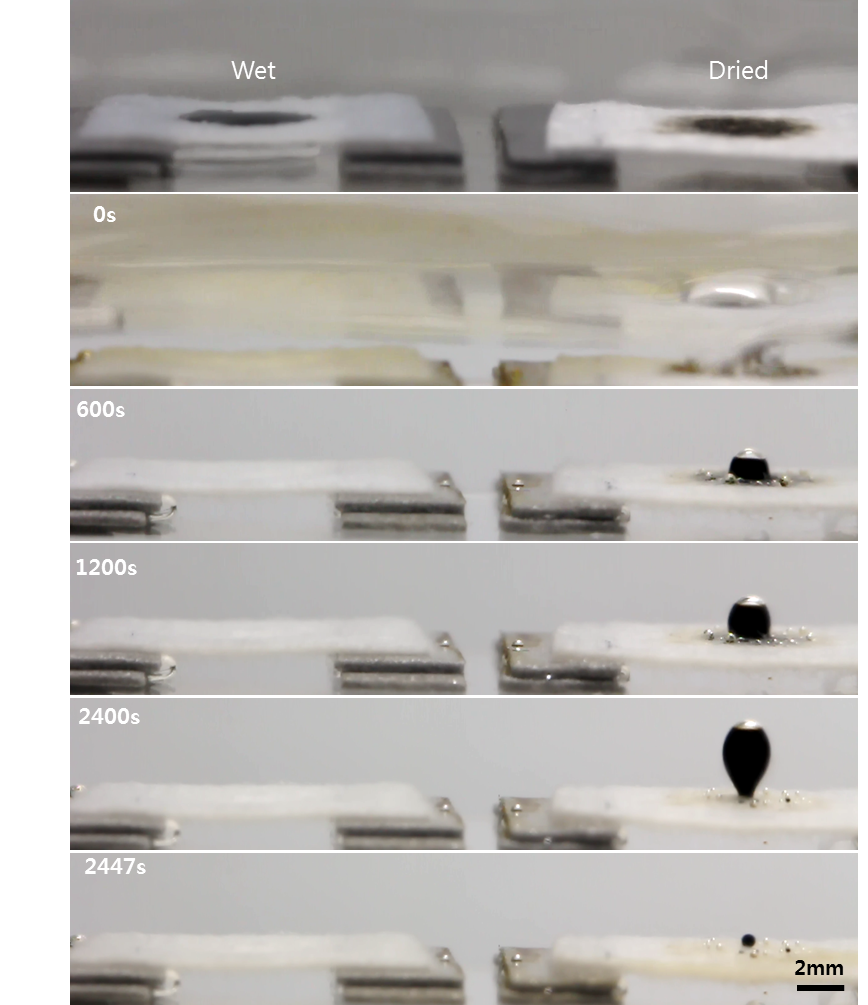


Figure S1. Oil-cleaning sequence on oil fouling under wet (left) and dry (right) states of fabric in water. Scale bar is 2 mm.

***
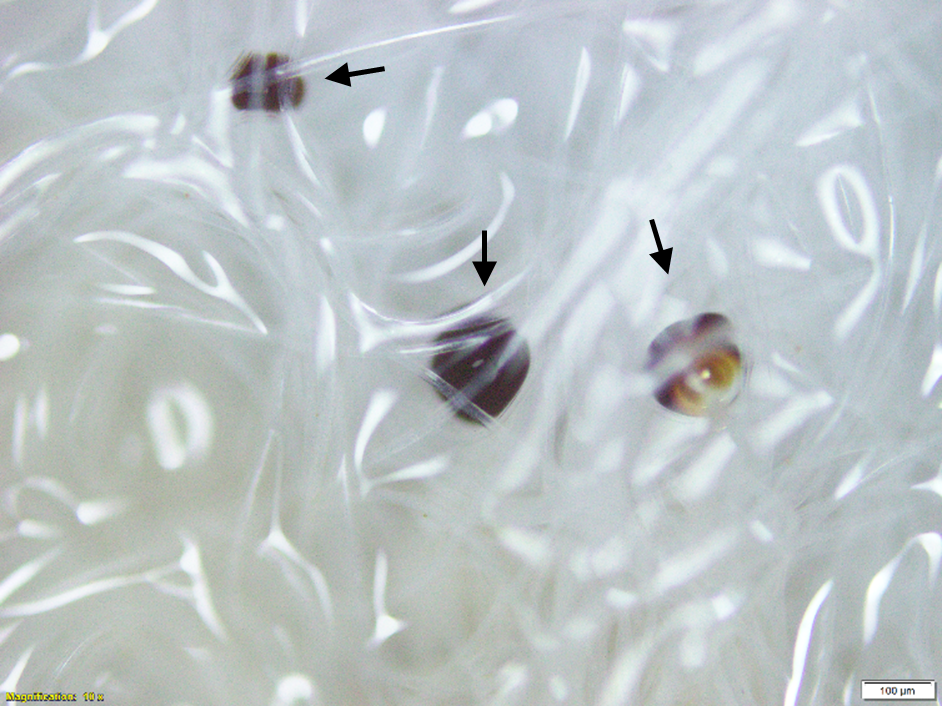
***

Figure S2. Optical microscopy image of oil trapped in rayon non-woven fabrics. The oil receded entirely and exhibited an almost spherical shape. However, it could not escape from the fabrics because adjacent or crossed fibers blocked the release of the trapped oil (arrowed).


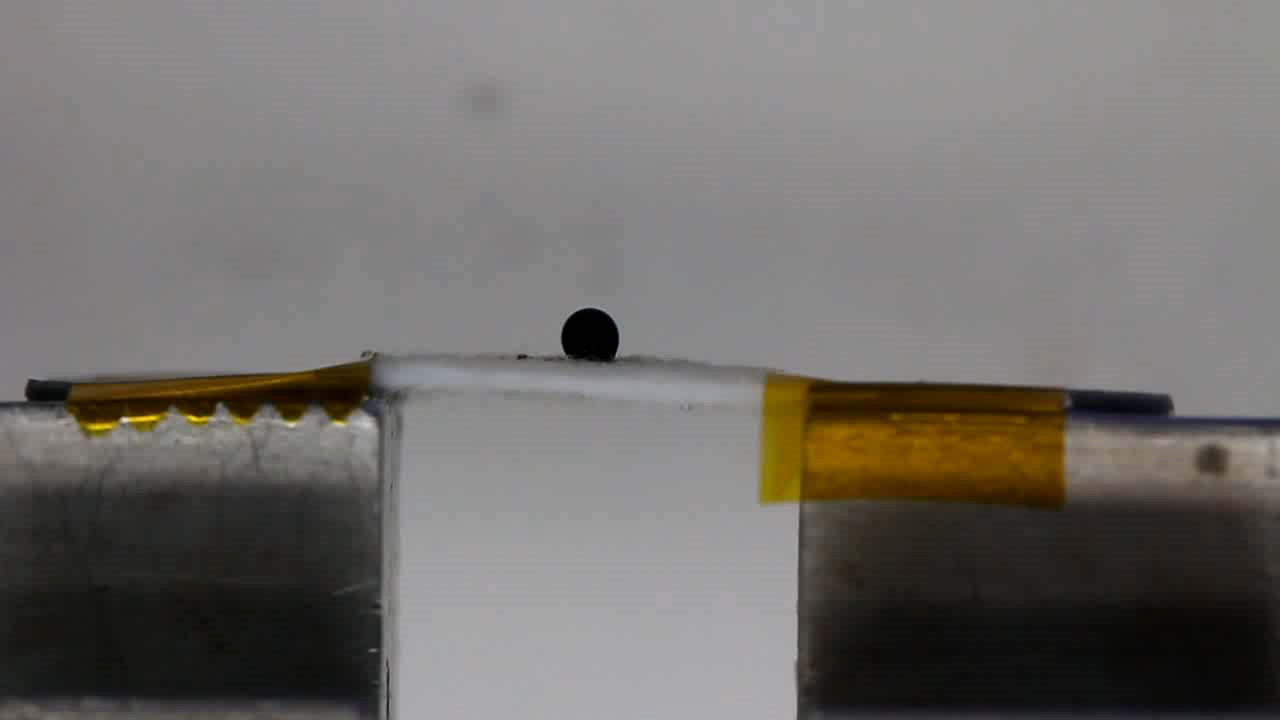


Movie S2. Extreme difference in contact angle hysteresis (CAH) between pristine and nanostructured surfaces. The oil droplet is strongly adhered to the pristine rayon even up to a sliding angle of ≈ 90°. In contrast, the oil on the nanostructured rayon is found to easily roll off at a sliding angle of ≈ 20°.


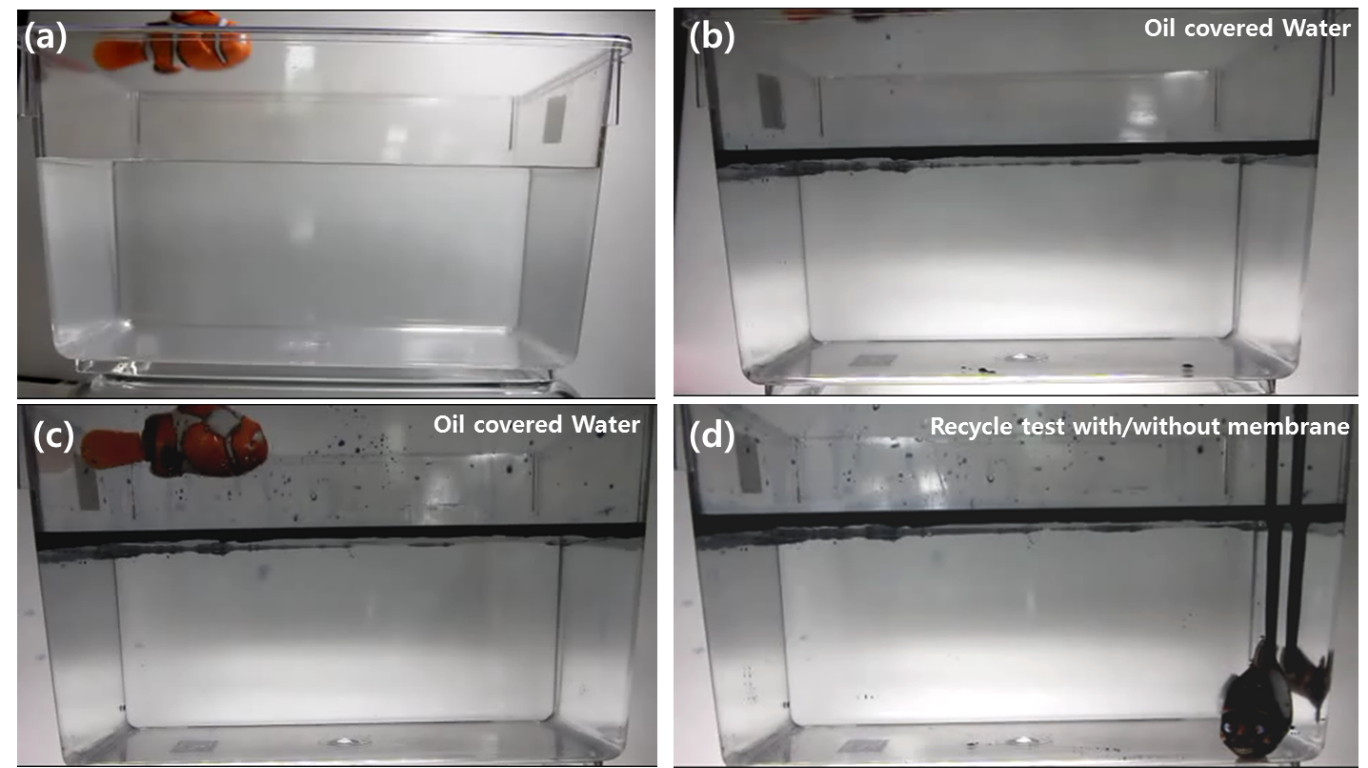


Movie S3. Four movie clips showing a swimming robot in water. Robot in (a) fresh and (b) oily water without any oil-proof fabric. The robot (c) immersed in oily water with nanostructured fabric (d) later removed from the fish.
